# Participation patterns in talent development in youth sports

**DOI:** 10.3389/fspor.2023.1175718

**Published:** 2023-05-18

**Authors:** Arne Güllich, Michael Barth, David Z. Hambrick, Brooke N. Macnamara

**Affiliations:** ^1^ Department of Sports Science, RPTU Kaiserslautern, Kaiserslautern, Germany; ^2^ Department of Sport Science, Universität Innsbruck, Innsbruck, Austria; ^3^ Department of Psychology, Michigan State University, East Lansing, MI, United States; ^4^ Department of Psychological Sciences, Case Western Reserve University, Cleveland, OH, United States

**Keywords:** youth sports, talent, performance, early specialization, deliberate practice, deliberate play

## Abstract

There has been a longstanding debate about the question: What amounts of what types of youth sport activities optimally facilitate later athletic excellence? This article provides a review of relevant research. We first evaluate popular conceptualizations of participation patterns—early specialization, deliberate practice, and deliberate play. Then, we review the available evidence on associations between performance and individual participation variables. The review reveals conceptual, definitional, and empirical flaws of the conceptions of early specialization, deliberate practice, and deliberate play. These approaches thus possess limited usefulness for empirical research. A review of studies considering individual, clearly defined participation variables provides a differentiated pattern of findings: Predictors of rapid junior performance and of long-term senior performance are opposite. Higher-performing juniors, compared to lower-performing peers, started playing their main sport, began involvement in talent promotion programs, and reached developmental performance milestones at younger ages, while accumulating larger amounts of coach-led main-sport practice, but less other-sports practice. In contrast, senior world-class athletes, compared to less-accomplished national-class peers, started playing their main sport, began involvement in talent promotion programs, and achieved performance milestones at older ages, while accumulating less coach-led main-sport practice, but more other-sports practice. We discuss implications for theory, practice, and future research.

## 1. Introduction

What types and amounts of sport activities optimally facilitate the achievement of athletic excellence? There is consensus that extensive sport-specific practice over multiple years is necessary. However, the question of optimal amounts of different types of sport activities in childhood and adolescence is the subject of a longstanding debate (1–4).

Participation patterns in youth sports have often been discussed in the context of the constructs of “*early specialization*” versus “*early diversification*” [e.g., (3, 4)]. Early specialization has commonly been associated with Ericsson et al.'s (5) proposed framework of “*deliberate practice*,” while early diversification has been associated with Côté et al.'s (3) proposal of childhood/adolescent multi-sport “*deliberate play*”.

In this article, we first evaluate the approaches of early specialization, deliberate practice, and deliberate play. Then, we review current empirical research addressing effects of participation variables on performance. Finally, we discuss implications for theory, practice, and future research.

## 2. Review of current research

### 2.1. Evaluation of the constructs of early specialization, deliberate practice, and deliberate play

Scientific research generally seeks to describe and explain laws of relationships between variables. Here, the focus is on relationships between childhood/adolescent participation variables and later performance. A youth athlete's participation pattern is composed of several participation variables, including age to begin playing their respective main sport, age to reach defined developmental performance milestones (e.g., first state, national, or international championships), types and number of sports they play, and amounts of organized coach-led practice and of informal peer-led play, both in their main sport and in other sports. These participation variables can all be measured individually as continuous, parametric variables, and their linear or non-linear associations with performance, and interactions with one another, can be quantified.

The construct of *early specialization* is problematic for research, primarily because it is not a sound scientific construct in several regards [for general issues of unfalsifiability of claims about early specialization, see e.g., (6, 7)].

1. There is no theoretically and/or empirically based definition of the construct [reviews in (8, 9)]. Instead, there are countless *ad hoc* definitions in the literature.
2. *Early* specialization has referred to varying age periods (6 years to late adolescence).
3. Early specialization has commonly been described as one composite construct composed of several constituents (9). These vary study to study to include, for example,
  (a) participation in intensive/extensive/increased hours of competitions/training and/or deliberate practice
  (b) that is/are specific/structured/systematic/targeted/focused/regular/intentional/purposeful/committed and/or effortful,
  (c) done year-round/over 8 or 6 months annually,
  (d) and done mainly/almost exclusively or exclusively, at the exclusion/reduction or limitation of deliberate play/other sports and/or other activities in general,
  (e) to achieve skill improvement/performance/athletic expertise/elite success or scholarships (9).

Most constituents lack operational definitions, and both the activity attributes and athletes’ motives (b, e) have typically been *ascribed* to the “specialized” activity, not empirically determined. Additionally, the early specialization composite construct and its constituents, although all continuous variables, have commonly been artificially dichotomized, dividing “specialized” versus “non-specialized” participants (9). These characteristics preclude the investigation of which individual participation variables are associated with performance and in which way [(7, 8), just as for other outcomes such as injuries or psychosocial wellbeing, e.g., (10, 11)]. Given that relevant participation variables can be recorded separately and as continuous variables, approaches at both forming one composite early specialization construct and its artificial dichotomization [or tripartition (10)] are neither necessary nor conducive to research (7).

Ericsson et al. (5) proposed that youth athletes should start *deliberate practice* at a young age and should subsequently maximize their amount of deliberate practice: individual sport-specific practice that is instructed and monitored by a coach, includes frequent repetition of a task, is done to improve one's performance, and is highly effortful and not inherently enjoyable. The authors partly *ascribed* activity attributes they deemed effective to performance (solitariness, effort, low enjoyment, performance motive) by way of synthetic *a priori* attribution (12) rather than empirical evidence [review in (13)]. Furthermore, athletes typically report high inherent enjoyment of practice activities that meet deliberate practice criteria, while their developmental sport engagement also includes extensive activities outside the original definition of deliberate practice: Team practice, playing forms, and competitions (13–19). Consequently, Ericsson (20) acknowledged that his conceptualization of deliberate practice has limited applicability to the sports domain.

In their proposal of *early diversification*, Côté et al. (3) suggested that youth athletes should delay increasing single-sport deliberate practice to the “investment stage” (16–18 years). This *late specialization* should be preceded by a “sampling stage” (6–12 years) and a “specialization stage” (13–15 years) with extensive *deliberate play* in multiple sports: Informal non-organized play that is regulated by the participants, rather than by a coach (i.e., peer-led), and is done for the inherent enjoyment of play, not for performance improvement (e.g., backyard soccer, street hockey, ice-hockey on a frozen lake). The authors distinguished deliberate play from other activities by several attributes (e.g., variability, time-on-task, motives, inherent enjoyment) and outcomes (skill transfer, future intrinsic motivation, prolonged engagement) *ascribed* by way of synthetic *a priori* attribution and extrapolation from general childhood non-sport play [for dissenting evidence from sports (13, 21–24)]. Furthermore, the age demarcations of Côté et al.'s (3) “stages” were normatively set rather than empirically determined and cannot take account of the great individual variation and gradual changes of different developmental sport activities through the course of an athletic career. In addition, given that age periods and amounts of each type of sport activity can be empirically recorded, an *a priori* normative categorization of career stages is unnecessary, but may constrict empirical research.

### 2.2. Effects of participation variables on performance

A commonality of the aforementioned approaches is that they *ascribed* participant motives, perceptions, and activity attributes to their composite constructs by way of *a priori* attribution or illegitimate extrapolation rather than empirical evidence. An alternative, appropriate research approach is to measure relevant, clearly defined participation variables separately—for example, athletes’ age to start playing their main-sport, age to reach developmental performance milestones, and age periods and amounts of organized coach-led practice and informal peer-led play, both in one's respective main sport and in other sports. This approach also has limitations; for example, it does not consider participants’ motives and perceptions [while these can also be integrated (13, 24)]. But its strengths include (a) the distinction of activity types considered critical in the aforementioned approaches by only the unambiguous criteria (the sport: main sport vs. other sports, and the setting: organized coach-led practice vs. informal peer-led play), and (b) enabling investigation of bivariate and potential multivariate interactive, linear and non-linear associations of performance with the individual participation variables. The approach would still allow for categorizations of participants, activity amounts, or career phases—but *a posteriori* based on the empirical data.

In a recent report (25), we systematically reviewed the findings from studies that have considered associations between achieved performance and these participation variables. Results of original studies have been inconsistent: Each of the participation variables was positively correlated with performance in some studies, but was uncorrelated or negatively correlated with performance in other studies. However, samples were heterogeneous in terms of athletes’ age category (juniors, seniors), performance levels (local to Olympic level), and types of sports.

To establish robust and generalizable findings, the available studies were synthesized in two recent meta-analyses (26, 27), structuring the findings from original studies by athletes’ age category (junior, senior), performance level (international, national, below), and types of sports. Analyses included 685 effect sizes from 131 studies with 9,241 athletes, 67% male, 33% female, 62% junior, and 38% senior athletes (i.e., competing in the highest, open-age category, typically in their 20–30 s); 1,003 athletes achieved international medals or top-ten placings and 4,818 competed at a national level.

Two questions were investigated:

1. Did higher- and lower-performing athletes differ in age to start playing their respective main sport, age to reach developmental performance milestones, and/or amounts of coach-led practice or peer-led play in either their main sport or in other sports?
2. Do effects of participation variables differ across athletes’ age category (juniors, seniors) or types of sports?

Central findings are summarized in Table 1. Participation variables predicted junior and senior performance. Moreover, childhood/adolescent participation variables differentiated later senior world-class and national-class athletes. However, predictors of early junior performance and of long-term senior performance were opposite.

**Table 1:** Meta-analytic mean effects (Cohen's $\bar{d}$) of participation variables on performance, separately for mean effects on junior performance overall (left column), senior performance overall (central column) and senior world-class vs. national-class athletes (right column).

Predictors	Effects on higher versus lower performance		
	Junior athletes	Senior athletes	Senior athletes
	Overall^a^	Overall^a^	WCl vs. NCl^b^
	$\bar{d}$	$\bar{d}$	$\bar{d}$
Age-related predictors			
Main sport starting age	−0.33**	0.28**	0.41**
Age to reach milestones^c^	−0.49**	0.36**	0.42**
Amount of activity throughout one's career			
Amount of coach-led practice			
In one's main sport	0.61**	0.20*	−0.23**
In other sports	−0.23**	0.47**	0.50**
Amount of peer-led play			
In one's main sport	0.24	0.17	−0.03
In other sports	−0.12*	0.13*	0.11
Amount of only early activity until age 15 years			
Amount of coach-led practice			
In one's main sport	0.53**	−0.10	−0.29**
In other sports	−0.14	0.51**	0.54**
Amount of peer-led play			
In one's main sport	0.18	0.14	0.03
In other sports^d^	—	0.15	0.14

Upper part: mean effects of activities accumulated throughout one's entire athletic career. Lower part: mean effects of only early activities accumulated until age 15 years. Based on data from Barth et al. (27). $\bar{d}$* *= meta-analytic mean Cohen's $\bar{d}$. Note the sign of effects for age- and activity-related predictors: a positive effect indicates that higher performance was associated with older (higher) ages and with greater activity amounts.

^a^ Comparisons of higher- and lower-performing athletes across all performance levels (international, national, regional level).

^b^ WCl, world class (international medalists or top ten), NCl, national class (national squad, top ten at national championships, national premier league).

^c^ E.g., first national championships, first international championships.

^d^ —, not enough effect sizes (*k* < 5) for juniors’ early other-sports peer-led play.

* Significance: *p* < .05.

** Significance: *p* < .01.

Overall, higher-performing juniors started playing their main sport at younger ages, achieved developmental performance milestones at younger ages, accumulated greater amounts of coach-led main-sport practice, and smaller amounts of other-sports practice, than lower-performing juniors (Table 1). In contrast, higher-performing senior athletes started playing their main sport at older ages, achieved developmental performance milestones at older ages, and accumulated greater amounts of coach-led other-sports practice, than lower-performing seniors. In addition, amount of coach-led main-sport practice was less predictive of senior performance than of junior performance, and senior performance was *unrelated* to *early* amount of main-sport practice (Table 1).

Senior world-class athletes started playing their main sport at older ages and achieved developmental performance milestones at older ages than their less-accomplished national-class counterparts. Relatedly, world-class athletes engaged in *less* coach-led main-sport practice, but more coach-led other-sports practice (Table 1). The senior world-class athletes practiced and competed in 1.9 other sports for 9.4 years, ending at age 18.1 years (sample-weighted means).

Although many athletes participated in considerable childhood/adolescent peer-led play—for example, senior world-class athletes’ total childhood/adolescent sport activity was 32% peer-led play (sample-weighted mean)—effects of peer-led play amounts, both main-sport and other-sports, on the differentiation between higher- and lower-performing athletes were negligible, both for junior and senior performance (Table 1).

The findings were robust across different types of sports [cgs sports (performance is measured in centimeters, grams, or seconds), game, combat, and artistic composition sports] (26, 27). Furthermore, central findings have been confirmed in multi-year prospective quasi-experiments, matched-pairs designs, and multivariate linear and non-linear analyses (28–32).

Finally, to fully understand the pattern of findings, three specific results from several original studies are relevant (28–41).

1. Senior world-class and national-class athletes had similar performance development until late adolescence and only diverged in early adulthood. The senior world-class athletes, compared to national-class counterparts, performed equivalent or less main-sport practice through the age interval. Therefore, childhood/adolescent multi-sport practice apparently had a delayed moderator effect via improved subsequent sport-specific *efficiency of practice*—i.e., performance improvement per practice amount.
2. The greater later performance improvement was rather based on better sport-specific perceptual-motor skill development than physical development (speed, power, endurance). This suggests that the improved sport-specific efficiency of practice primarily rested on better perceptual-motor *learning*.
3. The effect was not moderated by relatedness of an athlete's main sport with the other sports they played.

### 2.3. Effects of early involvement in talent promotion programs on performance

Talent promotion programs (TPPs) in youth sports seek to increase the long-term senior performance of talent-identified youth athletes (42, 43). They preferably select high-performing youth athletes and, once selected, attempt to further accelerate childhood/adolescent performance via expanded specialized practice, competitions, and corresponding environments and resources (high-profile coaching, facilities, athlete services) (42, 43). TPPs seek to involve identified talents at a young age, typically around puberty or younger, to enable a long period of TPP nurture until the anticipated age of peak performance.

Many of the selected early high performers have an early biological maturation [e.g., puberty, growth spurt (44)], have been born early within their birth-year [relative age effect (45)], and have already had large amounts of sport-specific training (27). The question arises whether younger TPP involvement is associated with higher performance in subsequent years.

Nineteen studies, involving 38 study samples from multiple sports and countries (29, 31, 36–39, 46–58), have investigated associations of athletes’ junior or senior performance with their age of beginning TPP involvement in terms of federations’ youth squads, selection teams, or sport academies. Table 2 reviews the findings. Consistent across performance levels and TPPs, higher-performing *juniors* were selected for TPPs at *younger* ages than lower-performing juniors. In contrast, higher-performing *seniors* were selected for TPPs at *older* ages than lower-performing seniors (Table 2).

**Table 2:** Mean effects (Cohen's $\bar{d}$) of the age of beginning involvement in talent promotion programs on early junior performance and on later senior performance.

Subsamples^a^	Effects on higher vs. lower performance	
	Junior athletes	Senior athletes
	$\bar{d}$	$\bar{d}$
Overall^b^	−0.60	0.61
World-class vs. national class^c^	−0.63	0.54
National class vs. regional class^c^	−0.50	0.67
Federation's squad/selection team^d^	−0.63	0.60
Youth sport academy^d^	−0.50	0.68

Junior athletes: *k* = 13, *N* = 1,674, senior athletes: *k* = 25, *N* = 5,400. $\bar{d}$ = sample-weighted mean Cohen's $\bar{d}$. Note the sign of effects: a negative effect indicates that higher performance was associated with a younger selection age, a positive effect indicates that higher performance was associated with an older selection age.

^a^ References (29, 31, 36–39, 46–58).

^b^ Pooled for federation's youth squad/selection team and youth sport academy and across performance levels.

^c^ Pooled for federation's youth squad/selection team and youth sport academy. World class = international medalists or top ten, national class = top ten at national championships or playing national premier league, regional = below.

^d^ Pooled across world-class, national, and regional performance levels.

## 3. Discussion

Investigating the association of performance with individual, unambiguous participation variables while distinguishing predictors of early junior performance and long-term senior performance provides a more differentiated pattern of findings than only considering task-specific deliberate practice or a composite, dichotomized early specialization construct. An early start, extensive coach-led main-sport practice with little or no other-sports practice, early TPP involvement, and rapid achievement of performance milestones appear to facilitate early junior performance. In contrast, a later start, reduced childhood/adolescent coach-led main-sport practice, more other-sports practice over more years, delayed TPP involvement, and delayed achievement of performance milestones appear to facilitate long-term senior world-class performance.

The findings do not call into question the importance of multi-year coach-led sport-specific practice and of juvenile performance progress. All the senior world-class and national-class athletes and high-performing junior athletes engaged in considerable main-sport practice and many had remarkable performance progress in their early years. However, athletes who had a particularly accelerated performance development in their early years—typically associated with increased main-sport practice, little or no other-sports practice, and early TPP involvement—are common among the highest junior performers and senior national-class athletes, but are rare among senior world-class athletes.

### 3.1. Theoretical implications

Traditional conceptions of deliberate practice, diversified deliberate play, as well as of giftedness (3, 5, 59), cannot adequately explain the full range of empirical observations concerning athletic performance, primarily because their central tenets are at odds with the empirical evidence. More specifically, they cannot explain the factors predicting the highest performance level, i.e., senior world class. Nor can they explain why predictors of short-term junior performance and long-term senior performance are opposite and why early non-specific practice facilitates later efficiency of sport-specific practice.

Alternatively, viewing youth sports participation through a neoclassical economic framework, especially the concepts of *efficiency* and *sustainability*, provides a fruitful heuristic to better understand the development into the highest athletic performance levels (26, 27, 30). In essence, as amounts of practice and competitions increase, *efficiency of practice* is paramount, because (1) resources are limited and must be economized (e.g., the athlete's time, body, load-tolerance, health), and (2) coaches and athletes seek to expand benefits (e.g., performance, enjoyment, prestige) while limiting costs (especially *opportunity costs*—the lost benefit of forgone other activities, such as time with family, friends, academics, hobbies, other sports) and risks (e.g., overtraining, injury, burnout). *Sustainability* is also paramount because (3) costs, risks, and benefits of participation patterns vary and may even be opposite regarding short- versus long-term outcomes.

Among high-level athletes who have all engaged in multi-year extensive sport-specific practice, the senior world-class athletes’ reduced main-sport practice combined with multi-year other-sports practice suggests a rather resource-preserving, cost-reducing, and risk-buffering childhood/adolescent investment pattern that yielded greater benefit in terms of performance in the long run. Practice and competition experiences in various sports diversify athletes’ “risk capital” and increase the odds that they find a sport that matches their talent and individual preferences [search and match theory (60, 61)]. Furthermore, childhood/adolescent multi-sport engagement has been reported to be associated with reduced risks of later overuse injuries and burnout (10, 11). Finally, the diverse learning experiences associated with practice and competitions in different sports may expand athletes’ *learning capital* for future long-term sport-specific perceptual-motor learning [theory of learning transfer as preparation for future learning, PFL (62)]. The varied learning experiences facilitate the athlete's ability to adapt to and exploit different learning opportunities and situations (63). The experiences with varying learning designs and methodologies also help the athlete understand individually more and less athlete-functional learning solutions (30, 62).

In contrast, intensified early main-sport practice with little or no other-sports practice implies reduced long-term benefit and expanded costs and risks for youth athletes. Relatedly, early TPP involvement may impose additional costs (expanded time demands from additional training, competitions, athlete services, transit times) and risks (overtraining, later overuse injuries) on the youth athlete. In addition, there may be two specific selection effects, in that athletes who have an accelerated biological maturation [puberty, growth spurt (44)] and are relatively old within their birth year [relative age effect (45)] have a performance advantage during adolescence which, however, diminishes or is even reversed by adulthood (64–66).

### 3.2. Practical implications

Youth sport programs should seek to limit youth athletes’ costs and risks while maximizing their benefits. The empirical evidence suggests three clear practical implications.

1. Youth sport coaches and managers make a choice that may be poorly- or well-informed: To reinforce rapid junior success at the expense of long-term senior success or to facilitate long-term senior success at the expense of early junior success. To facilitate long-term senior success (and youth athletes’ physical and psychological wellbeing), youth coaches should avoid excessive specialized single-sport practice and encourage youth athletes and provide opportunities to practice and compete in 1–2 other sports.
2. Given that particularly early TPP involvement is negatively correlated with long-term senior performance, TPPs should postpone selection to later ages. In addition, aiming to select the youth athletes with the greatest future potential, talent selection should consider their participation history in terms of moderate sport-specific training with multi-sport practice prior to selection.
3. Evaluating the work of youth coaches and TPPs by their youth athletes’ early junior performance may elicit dysfunctional incentives. Rather, it is functional to evaluate their work by the performance progress the youth athletes make in subsequent years into adulthood.

### 3.3. Future research directions

Factors that make the difference among the highest athletic performance levels—senior world-class and national-class performance—cannot be inferred by extrapolating findings from junior athletes, lower performance levels, or extreme contrast comparison [such as international versus local level, e.g., (19, 33, 67–70)]. To predict the highest performance levels, the goal for future research is to further investigate childhood/adolescent participation factors of the highest-performing senior athletes. The economic concepts of *efficiency* and *sustainability* provide a fruitful heuristic, and lead to three questions:

1. What short- and long-term, material and immaterial costs, risks, and benefits do different childhood/adolescent participation patterns yield?
2. What objective and subjective value does each of the costs, risks, and benefits have?
3. What is the eventual ratio of the summed value of all benefits relative to the summed value of all costs and risks emerging from different childhood/adolescent participation patterns?

This research will advance an economic theory of the development of athletic excellence, and contribute to a well-substantiated scientific foundation for designing youth sport programs.
